# Effectiveness of COVID-19 Vaccines in Preventing SARS-CoV-2 Infection Among Frontline Workers Before and During B.1.617.2 (Delta) Variant Predominance — Eight U.S. Locations, December 2020–August 2021

**DOI:** 10.15585/mmwr.mm7034e4

**Published:** 2021-08-27

**Authors:** Ashley Fowlkes, Manjusha Gaglani, Kimberly Groover, Matthew S. Thiese, Harmony Tyner, Katherine Ellingson, HEROES-RECOVER Cohorts

**Affiliations:** ^1^CDC COVID-19 Response Team; ^2^Baylor Scott and White Health, Texas A&M University College of Medicine, Temple, Texas; ^3^Abt Associates, Inc., Rockville, Maryland; ^4^University of Utah, Salt Lake City, Utah; ^5^St. Luke’s Regional Health Care System, Duluth, Minnesota; ^6^Mel and Enid Zuckerman College of Public Health, University of Arizona, Tucson, Arizona.

During December 14, 2020–April 10, 2021, data from the HEROES-RECOVER Cohorts,* a network of prospective cohorts among frontline workers, showed that the Pfizer-BioNTech and Moderna mRNA COVID-19 vaccines were approximately 90% effective in preventing symptomatic and asymptomatic infection with SARS-CoV-2, the virus that causes COVID-19, in real-world conditions (*1*,*2*). This report updates vaccine effectiveness (VE) estimates including all COVID-19 vaccines available through August 14, 2021, and examines whether VE differs for adults with increasing time since completion of all recommended vaccine doses. VE before and during SARS-CoV-2 B.1.617.2 (Delta) variant predominance, which coincided with an increase in reported COVID-19 vaccine breakthrough infections, were compared (*3*,*4*).

Methods for the HEROES-RECOVER Cohorts have been published previously (*1*,*2*,*5*). Health care personnel, first responders, and other essential and frontline workers in eight U.S. locations across six states were tested weekly for SARS-CoV-2 infection by reverse transcription–polymerase chain reaction (RT-PCR)^†^ and upon the onset of any COVID-19–like illness. Weeks when the Delta variant accounted for ≥50% of viruses sequenced, based on data from each respective location, were defined as weeks of Delta variant predominance. Vaccination was documented by self-report and verified by provision of vaccine cards or extraction from electronic medical records or state immunization registries. Among 4,217 participants, 3,483 (83%) were vaccinated; 2,278 (65%) received Pfizer-BioNTech, 1,138 (33%) Moderna, and 67 (2%) Janssen (Johnson & Johnson) COVID-19 vaccines. Cox proportional hazards models were used to calculate ratios of unvaccinated to fully vaccinated (≥14 days after receipt of all recommended COVID-19 vaccine doses) infection rates, adjusted for occupation, site, and local viral circulation (*6*), and weighted for inverse probability of vaccination using sociodemographic characteristics, health information, frequency of close social contact, and mask use. This activity was reviewed by CDC and was conducted consistent with applicable federal law and CDC policy.^§^

During the 35-week study period, 4,136 participants with no previous laboratory-documented SARS-CoV-2 infection contributed a median of 20 unvaccinated days per participant (interquartile range [IQR] = 8–45 days; total = 181,357 days), during which 194 SARS-CoV-2 infections were identified; 89.7% of these infections were symptomatic. A total of 2,976 participants contributed a median of 177 fully vaccinated days (IQR = 115–195 days; total = 455,175 days) with 34 infections, 80.6% of which were symptomatic. Adjusted VE against SARS-CoV-2 infection was 80% (95% confidence interval [CI] = 69%–88%). The VE point estimate was 85% among participants for whom <120 days had elapsed since completion of full vaccination compared with 73% among those for whom ≥150 days had elapsed; however the VE 95% CI were overlapping, indicating the difference was not statistically significant (Table).

**TABLE:** Effectiveness of COVID-19 vaccines against any SARS-CoV-2 infection among frontline workers, by B.1.617.2 (Delta) variant predominance and time since full vaccination — eight U.S. locations, December 2020–August 2021

Period and vaccination status	No. of contributing participants*	Total no. of person-days	Median days (IQR)	No. of SARS-CoV-2 infections	Adjusted VE,^†^ % (95% CI)
**Full cohort to date**					
Unvaccinated	4,136	181,357	20 (8–45)	194	N/A
Fully vaccinated^§^	2,976	454,832	177 (115–195)	34	80 (69–88)
14–119 days after full vaccination	2,923	284,617	106 (106–106)	13	85 (68–93)
120–149 days after full vaccination	2,369	66,006	30 (30–30)	3	81 (34–95)
≥150 days after full vaccination	2,129	104,174	52 (37–64)	18	73 (49–86)
**Pre-Delta variant predominance**					
Unvaccinated	4,137	156,626	19 (8–43)	175	N/A
Fully vaccinated	2,875	329,865	124 (95–149)	10	91 (81–96)
**Delta variant predominance**					
Unvaccinated	488	24,871	43 (37–69)	19	N/A
Fully vaccinated	2,352	119,218	49 (35–56)	24	66 (26–84)

**Abbreviations:** CI = confidence interval; IQR = interquartile range; N/A = not applicable; SMD = standardized mean difference; VE = vaccine effectiveness.

* Person-days between the date of any dose of COVID-19 vaccine and fully vaccinated status were excluded from VE models because of indeterminate immune status. Participants with SARS-CoV-2 infection during this period were also excluded; in the pre-Delta period, 47 participants were excluded, and in the Delta period, two participants were excluded. Contributing participants in vaccination categories also do not equal the total number of participants in the cohort.

^†^ Adjusted VE was inversely weighted for probability of being vaccinated and adjusted for local virus circulation, study location, and occupation. Delta variant models were additionally adjusted for ethnicity. All Cox regression models met the proportional hazards assumption. To calculate the probability of being vaccinated for each period, boosted regression models were fit including covariates for site, sociodemographic characteristics, health information, frequency of close social contact, mask use, and local virus circulation. In the full cohort to date and the pre-Delta cohort, all covariates met balance criteria of SMD<0.2 after weighting except mask use at work (SMD = 0.227 and 0.207, respectively) but was not found to change VE estimates by ≥3% when added to the models. In the Delta predominant cohort occupation, ethnicity, influenza vaccination, and mask use at work did not meet balance criteria (SMD range = 0.206–0.288); influenza vaccination and mask use at work did not change VE estimates by ≥3%; however, occupation and ethnicity did change VE by ≥3% and were therefore included as covariates in the Cox regression model for VE.

^§^ Fully vaccinated was defined as ≥14 days after receipt of all recommended COVID-19 vaccine doses.

During Delta variant–predominant weeks at study sites, 488 unvaccinated participants contributed a median of 43 days (IQR = 37–69 days; total = 24,871 days) with 19 SARS-CoV-2 infections (94.7% symptomatic); 2,352 fully vaccinated participants contributed a median of 49 days (IQR = 35–56 days; total = 119,218 days) with 24 SARS-CoV-2 infections (75.0% symptomatic). Adjusted VE during this Delta predominant period was 66% (95% CI = 26%–84%) compared with 91% (95% CI = 81%–96%) during the months preceding Delta predominance.

During December 14, 2020–August 14, 2021, full vaccination with COVID-19 vaccines was 80% effective in preventing RT-PCR–confirmed SARS-CoV-2 infection among frontline workers, further affirming the highly protective benefit of full vaccination up to and through the most recent summer U.S. COVID-19 pandemic waves. The VE point estimates declined from 91% before predominance of the SARS-CoV-2 Delta variant to 66% since the SARS-CoV-2 Delta variant became predominant at the HEROES-RECOVER cohort study sites; however, this trend should be interpreted with caution because VE might also be declining as time since vaccination increases and because of poor precision in estimates due to limited number of weeks of observation and few infections among participants. As with all observational VE studies, unmeasured and residual confounding might be present. Active surveillance through the cohort is ongoing and VE estimates will be monitored continuously. Although these interim findings suggest a moderate reduction in the effectiveness of COVID-19 vaccines in preventing infection, the sustained two thirds reduction in infection risk underscores the continued importance and benefits of COVID-19 vaccination.

## Footnotes

* Arizona Healthcare, Emergency Response and Other Essential Workers Surveillance Study (HEROES) conducted in Phoenix, Tucson, and other noncentrally located areas in Arizona; Research on the Epidemiology of SARS-CoV-2 in Essential Response Personnel (RECOVER) conducted in Miami, Florida; Duluth, Minnesota; Portland, Oregon; Temple, Texas; and Salt Lake City, Utah.

† RT-PCR was conducted using the Quidel Lyra SARS-CoV-2 Assay (before November 2020) or TaqPath COVID-19 Combo Kit (Applied Biosystems) at the Marshfield Clinic Research Institute (Marshfield, WI).

§ 45 C.F.R. part 46.102(l)(2), 21 C.F.R. part 56; 42 U.S.C. Sect 241(d); 5 U.S.C. Sect 552a; 44 U.S.C. Sect 3501 et seq.
